# Association of *Filifactor alocis* and its RTX toxin gene *ftxA* with periodontal attachment loss, and in synergy with *Aggregatibacter actinomycetemcomitans*

**DOI:** 10.3389/fcimb.2024.1376358

**Published:** 2024-03-26

**Authors:** Zeinab Razooqi, Ingeborg Tjellström, Carola Höglund Åberg, Francis Kwamin, Rolf Claesson, Dorte Haubek, Anders Johansson, Jan Oscarsson

**Affiliations:** ^1^ Department of Odontology, Umeå University, Umeå, Sweden; ^2^ Dental School University of Ghana, Korle-Bu, Accra, Ghana; ^3^ Jammerbugt Municipal Dental Service, Brovst, Denmark

**Keywords:** *Filifactor alocis*, FtxA, RTX toxin, *Aggregatibacter actinomycetemcomitans*, JP2, periodontitis, clinical attachment loss (CAL)

## Abstract

The Gram-positive bacterium, *Filifactor alocis* is an oral pathogen, and approximately 50% of known strains encode a recently identified repeat-in-toxin (RTX) protein, FtxA. By assessing a longitudinal Ghanaian study population of adolescents (10-19 years of age; mean age 13.2 years), we recently discovered a possible correlation between deep periodontal pockets measured at the two-year follow-up, presence of the *ftxA* gene, and a high quantity of *F. alocis*. To further understand the contribution of *F. alocis* and FtxA in periodontal disease, we used qPCR in the present study to assess the carriage loads of *F. alocis* and the prevalence of its *ftxA* gene in subgingival plaque specimens, sampled at baseline from the Ghanaian cohort (n=500). Comparing these results with the recorded clinical attachment loss (CAL) longitudinal progression data from the two-year follow up, we concluded that carriers of *ftxA*-positive *F. alocis* typically exhibited higher loads of the bacterium. Moreover, high carriage loads of *F. alocis* and concomitant presence of the *ftxA* gene were two factors that were both associated with an enhanced prevalence of CAL progression. Interestingly, CAL progression appeared to be further promoted upon the simultaneous presence of *F. alocis* and the non-JP2 genotype of *Aggregatibacter actinomycetemcomitans*. Taken together, our present findings are consistent with the notion that *F. alocis* and its *ftxA* gene promotes CAL during periodontal disease.

## 1 Introduction

Periodontitis is a bacterial-induced oral inflammatory disease, which over time degrades the periodontal tissues, ultimately causing tooth loss (Pihlstrom et al., 2005). Research is indicating that when the natural balance among the resident bacterial species is disturbed, causing a dysbiotic environment, some of the bacteria might exhibit potential pathogenic characteristics, leading to an inflammatory response in the periodontal tissues, which together with factors related to the host susceptibility promotes the degradation (Darveau, 2010). Periodontal diseases affecting adolescents and young individuals typically have been typically referred to as aggressive periodontitis and are at present defined as grade C indicating a rapid progress (Tonetti et al., 2018). There is a wide geographical spread (Bouziane et al., 2020). Among adolescents from the western and northern parts of Africa, the disease has been observed to have a high incidence (Kissa et al., 2022; Yoshida et al., 2021). A highly leukotoxin (LtxA)-producing genotype of the Gram-negative bacterium, *Aggregatibacter actinomycetemcomitans*, JP2, is associated with the periodontal disease progress in these geographical regions (Haubek et al., 2008; Höglund Åberg et al., 2014). It carries a typical deletion of 530 base pairs (bp) in the *ltxCABD* promoter (Brogan et al., 1994). To the best of our knowledge, few studies executed in the northern and western geographical regions of Africa have been reported on regarding the relation between periodontitis and the progression of this disease in young individuals, and concomitantly focusing on bacterial species other than *A. actinomycetemcomitans* (Yoshida et al., 2021; Dahlén et al., 2014), thus including the Gram-positive, anaerobic bacterium *Filifactor alocis*. This species has recently been identified in the oral microbiome by the method called high throughput DNA sequencing. *F. alocis* is culturable and is regarded as an emerging oral pathogen with significant roles in the etiology of periodontal (Aruni et al., 2014; Greenwood et al., 2020), peri-implantitis (Sanz-Martin et al., 2017), and endodontic (Zehnder et al., 2017) infections. It is a potential biomarker for active disease in young children (Aruni et al., 2014). Interestingly, *A. actinomycetemcomitans* and *F. alocis* seem to have a synergistic relationship in active periodontal disease (Fine et al., 2013; Wang et al., 2013). However, *A. actinomycetemcomitans* appears thereafter to be outcompeted by *F. alocis* in the deeper pockets (Dahlén et al., 2014). This suggests that *F. alocis* has mechanisms to be able to adapt and drive the disease process forward in the absence of *A. actinomycetemcomitans*. Mechanisms for this are yet unknown.

In the present work, we have assessed longitudinal data from a study population of adolescents in Ghana. This study population was followed up after two years (Höglund Åberg et al., 2014; Höglund Åberg et al., 2012). In the previous studies, cultivation and PCR were used to analyse the pooled subgingival plaque samples, which revealed a 54.2% prevalence of *A. actinomycetemcomitans* at baseline (Höglund Åberg et al., 2012). Among the individuals, it was also found that 8.8% in the studied population carried the JP2 genotype of *A. actinomycetemcomitans* (Höglund Åberg et al., 2012). At baseline, an association between clinical attachment loss (CAL) and carriage of *A. actinomycetemcomitans* was observed, and in addition an increased extent regarding individuals carrying the JP2 genotype (Höglund Åberg et al., 2012).

*F. alocis* was recently found to encode and express a putative repeats-in-toxin (RTX) protein, FtxA (Bao et al., 2022; Oscarsson et al., 2020). Interestingly, only approximately 50% of the isolated *F. alocis* strains appeared to carry the *ftxA* gene (Oscarsson et al., 2020), suggesting the presence of a potentially more virulent genotype of this species. In a recent study (Razooqi et al., 2022), a possible correlation between deep periodontal pockets, presence of the *ftxA* gene, and a high quantity of *F. alocis* in the Ghanaian study population could be seen. Moreover, there seemed to be an association between the presence of the *ftxA* gene, the JP2 genotype of *A. actinomycetemcomitans*, high levels of *F. alocis*, and deep periodontal pockets (Razooqi et al., 2022).

To further understand the potential contribution of *F. alocis* and its *ftxA* gene in periodontal disease, we aimed at determining the loads of carried *F. alocis* at baseline among the individuals in the Ghanaian study cohort, and the prevalence of *ftxA*, and relate these data to the presence and/or absence, respectively of periodontal attachment loss progress observed at the two-year follow-up.

## 2 Materials and methods

### 2.1 Study population

All clinical specimens used in the present work were earlier collected from adolescents (10-19 years of age; mean age 13.2 years) going to 11 Ghanaian schools, and consisted of subgingival plaque samples from nine different sites of periodontal pockets collected with paper points as described (Höglund Åberg et al., 2012). These samples were divided into three groups, referred to as A, B and C. Plaque in group A originated from mesial pockets of the first permanent molars, group B from the distal pockets of the permanent central incisors, and in group C plaque was sampled from one specific individually chosen site. A total of 500 adolescents were included at baseline (Höglund Åberg et al., 2012). At the two-year-follow-up, 397 of the same 500 adolescents could be included, and the clinical recordings were the same as for baseline, *i.e.*, including CAL. Individuals with at least one site with CAL 3 mm or more were classified as CAL-positive. If CAL increased 3 mm or more in at least one site from baseline to the two-year follow-up observations, the individual was classified as ‘CAL progression positive’ (Höglund Åberg et al., 2014). Presence of *A. actinomycetemcomitans* and its JP2 genotype, respectively in the baseline samples were also determined earlier (Höglund Åberg et al., 2014).

### 2.2 DNA extraction

The plaque samples, A, B and C, were pooled, *i.e.*, mixed in equal proportions and subject to genomic DNA isolation using a GXT NA Extraction Kit^®^ (Hain Lifesience, GmBH, Nehren, Germany) and an Arrow extraction instrument (Diasorin, Dublin, Ireland) using procedures described earlier (Razooqi et al., 2022).

### 2.3 Quantitative PCR analysis of *F. alocis*

For this procedure, DNA isolated from the subgingival plaque samples, A, B and C, were mixed in equal proportions, and served as templates in qPCR for each of the individuals (n=500), respectively. The KAPA SYBR^®^ FAST qPCR Kit (Kapa Biosystems, Wilmington, MA, USA) was used, with cycling conditions as described previously (Razooqi et al., 2022), and with a forward (5’-AGGCAGCTTGCCATACTGCG-3’), and a reverse (5’-ACTGTTAGCAACTACCGATGT-3’) oligonucleotide primer, respectively, targeting the *F. alocis* 16s rRNA gene (Siqueira and Rocas, 2003). Loads of *F. alocis* were determined as numbers of cells/ml sample as described (Razooqi et al., 2022).

### 2.4 PCR determination of presence of the *ftxA* gene of *F. alocis*

A forward (5′-GGCTCAGATACCTACTTCTTC-3′) and a reverse (5′-GAAGGCTATGATTTGATTGTTTCC-3′) oligonucleotide primer were used to amplify a 798-base pair (bp) internal fragment of the *ftxA* gene, as described previously (Oscarsson et al., 2020).

### 2.5 Statistical analysis

Data analyses were performed using SPSS 22.0 (SPSS Inc., Chicago, IL, USA), or Microsoft Excel (version 16.80). Statistical parameters included those earlier recorded regarding the presence or non-presence of (i) CAL for the individuals at baseline, (ii) progression of CAL recorded at the two-year follow up, (iii) *A. actinomycetemcomitans*, and (iv) the JP2 genotype of *A. actinomycetemcomitans*, respectively. Statistical parameters also included the microbial data at baseline deduced in the present work, *i.e.*, presence or non-presence of *F. alocis* (v), the *ftxA* gene (vi), and load of *F. alocis* (vii). Significant differences between sample groups were examined with the Mann–Whitney U test or *t*-test. Results were estimated by an odds ratio (OR) with 95% confidence interval (CI).

### 2.6 Ethics statement

The studies in the present work were ethically approved by Medical Research, University of Ghana (IRB 000 1276), and from the local Ethical committee of Umeå University, Sweden (Dnr 2010-188-31M).

## 3 Results

### 3.1 Prevalence and carriage levels of *F. alocis*

**Figure 1:**
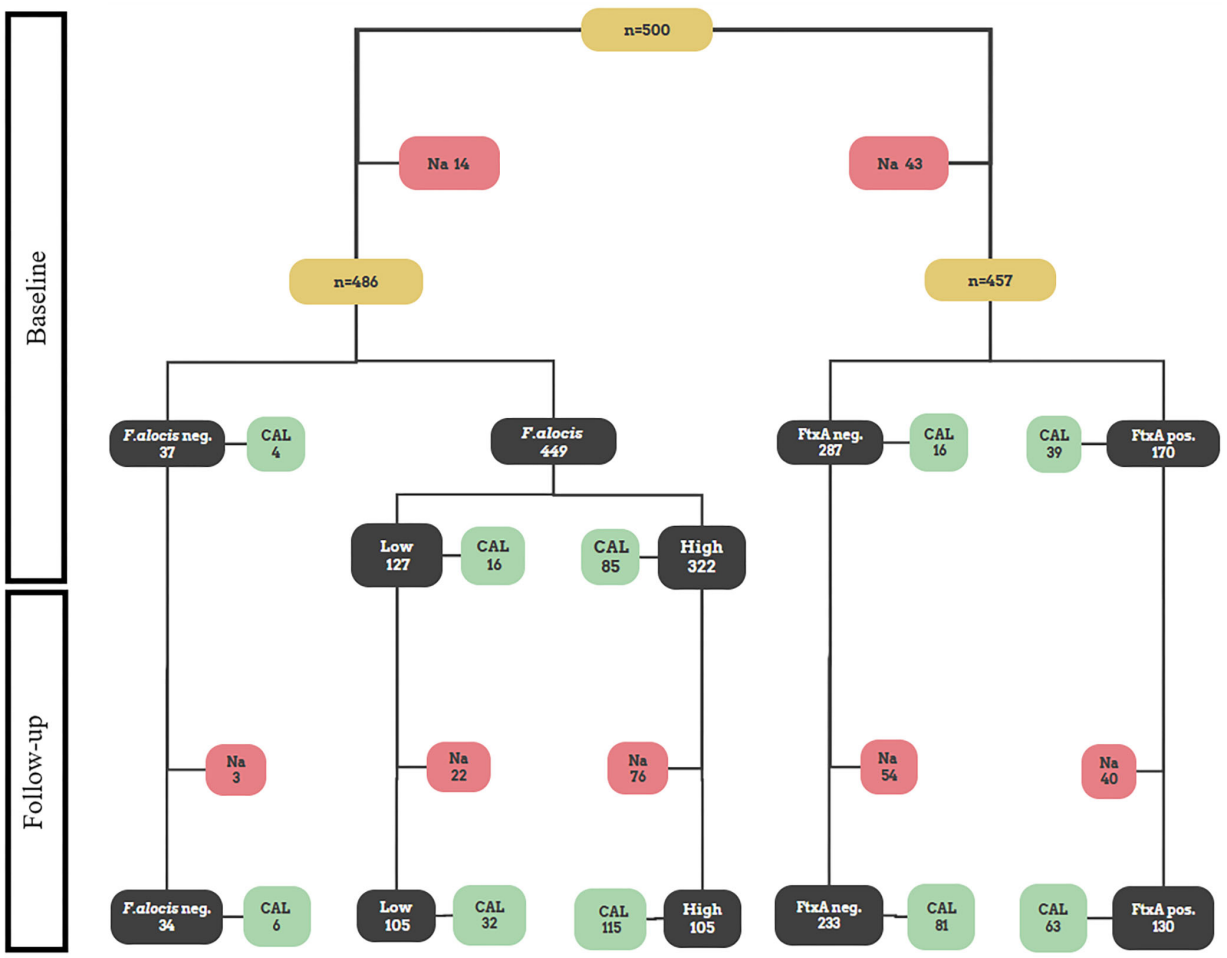
Flowchart illustrating a schematic outline of the present study. All figures in the flowchart represent numbers of patients. Pooled subgingival plaque samples from 500 Ghanaian adolescents were originally sampled at baseline for subsequent analysis of microbial data, and CAL was measured (Höglund Åberg et al., 2012). Out of those, 486 were assessed in the present work regarding presence of high and low levels of *F. alocis*, respectively, and 457 for presence or absence of the *ftxA* gene, respectively. Thereafter, at the two-year follow up, the re-examined individuals (n=385) were assessed regarding CAL, and for CAL progression relative to baseline (Höglund Åberg et al., 2014). Indicated are also numbers of non-analyzed samples (Na), which represent samples that were not analyzed due to lack of material.

A flow chart summarizing the outline of the present study is shown in **Figure 1**. For each of the individuals (n=500), a pooled subgingival plaque sample collected at baseline was analyzed with regards to *F. alocis* loads by qPCR. At baseline, *F*. *alocis* was found to be carried by 486 out of the 500 analyzed individuals, *i.e.*, a prevalence of 97.2%. Absence of *F. alocis* (i.e., *F. alocis*-negative) was based on a detection level <100 cells/ml sample. Cut off values of 10,000 *F. alocis* cells/ml sample was then used to divide the individuals as carriers of “high” (≥10,000), and “low” (100<10,000) loads of *F. alocis* per ml sample, respectively. Out of the 486 individuals carrying *F. alocis*, 322 (66.3%) were revealed to carry high, and 164 (33.7%) low levels of this bacterium, respectively (**Table 1**).

**Table 1:** Frequency (%) of carriers with CAL among the individuals carrying *A. actinomycetemcomitans* (*Aa*) and its JP2 or non-JP2 genotype, and *F. alocis* (*Fa*) at low and high loads, and Fa that is *ftxA* (positive; pos) or without *ftxA* (negative; neg), respectively.

	Baseline		Follow up		Progression
Carrier	Frequency of carrier (%)	Carriers with CAL (%)	Frequency of carrier (%)	Carriers with CAL (%)	Carriers with CAL (%)
No Aa^1)^	229/500 (45.8)	33/229 (14.4)	190/397 (47.9)	37/190 (19.5)	24/190 (12.6)
Non-JP2^1)^	227/500 (45.4)	64/227 (28.2)	169/397 (42.6)	88/169 (52.1)	62/169 (36.7)
JP2^1)^	44/500(8.0)	10/44 (22.7)	38/397 (9.6)	31/38 (81.6)	27/38(71.1)
Fa<10^2^	37/486(7.6)	4/37(10.8)	34/385(8.8)	6/34(17.6)	1/34(2.9)
10^2^ <Fa<10^4^	127/486 (33.7)	16/127 (12.2)	105/385 (36.1)	32/105 (27.3)	19/105 (18.1)
Fa ≥10^4^	322/486 (66.3)	85/322 (26.4)	246/385 (63.9)	115/246 (46.7)	92/246 (37.4)
*ftxA* neg	287/457 (62.8)	51/287 (17.8)	233/363 (64.2)	81/233 (34.8)	56/233 (24.0)
*ftxA* pos	170/457 (37.2)	39/170 (22.9)	130/363 (35.8)	63/130 (48.5)	50/130 (38.5)

^1)^Determined earlier (Höglund Åberg et al., 2012).

Frequency (%) of individuals with CAL was deduced at baseline (≥3 mm), follow up (≥3 mm), and progression (≥3 mm).

### 3.2 High carriage levels of *F. alocis* are associated with enhanced prevalence of CAL progression

**Figure 2:**
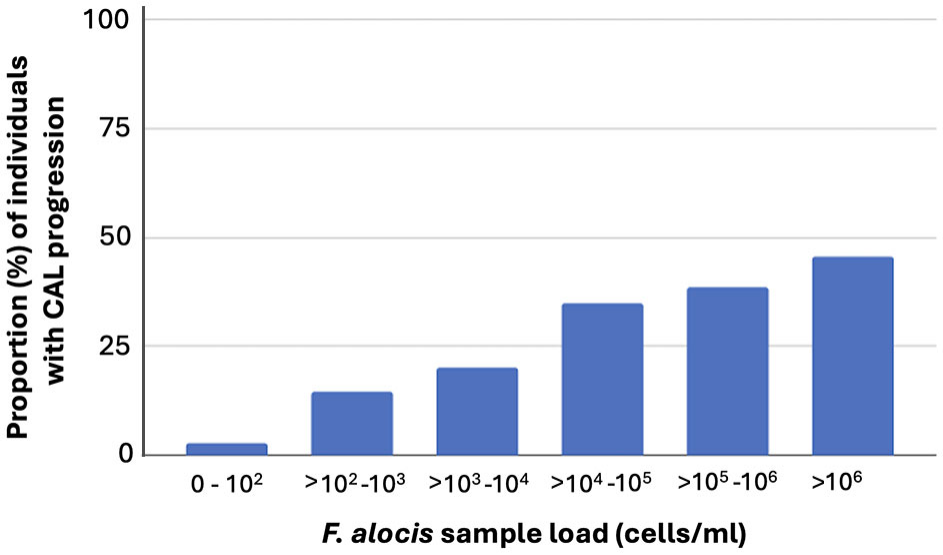
Prevalence (%) of CAL progression among the 385 individuals assessed at the follow up, related to their carriage levels of *F. alocis*, determined at baseline, in pooled subgingival plaque samples.

As summarized in **Table 1**, at the follow up (n=385), there were 246 (63.9%) individuals who carried high levels of *F. alocis* at the baseline examination. Of these 246, 46.7% exhibited CAL, and 37.4% a progressed CAL at the follow up relative to baseline. The prevalence of CAL progression was also gradually enhanced relative to increased carriage loads of *F. alocis* (**Figure 2**). As summarized in **Table 2**, relative to the low-level and non-carriers of *F. alocis* as grouped together (<10^4^ cells/ml sample), those carrying high loads (≥10^4^ cells/ml sample) exhibited a significantly increased prevalence of CAL both at baseline (OR = 2.58; *p*<0.001), and at follow up (OR = 2.33; *p*<0.001), and also regarding progression of CAL (OR = 3.56; *p*<0.001).

**Table 2:** Sample group comparisons (target versus reference) as outlined below, related to odds ratio for CAL at baseline (BL ≥3 mm), follow up (FU ≥ 3 mm), and progression (BL-FU ≥3 mm).

		Baseline		Follow up		Progression	
Target	Reference	OR (± 95% CI)	*p*-value	OR (± 95% CI)	*p*-value	OR (± 95% CI)	*p*-value
Non-JP2	Aa-neg	2.33 (1.46-3.73)	<0.001*	4.49 (2.81-7.18)	<0.001*	4.01 (2.36-6.81)	<0.001*
JP2	Aa-neg	1.75 (0.79-3.87)	0.169	18.31 (7.48-44.83)	<0.001*	16.98 (7.47-38.60)	<0.001*
Fa ≥10^4^	Fa <10^4^	2.58 (1.52-4.38)	<0.001*	2.33 (1.49-3.66)	<0.001*	3.56 (2.07-6.10)	<0.001*
*ftxA* pos	*ftx*A -	1.38 (0.86-2.20)	0.180	1.77 (1.14-2.73)	0.011*	1.96 (1.24-3.14)	0.004*

(* = statistically significant; *p*<0.05).

### 3.3 Presence of the *ftxA* gene is associated with higher loads of *F. alocis*

**Figure 3:**
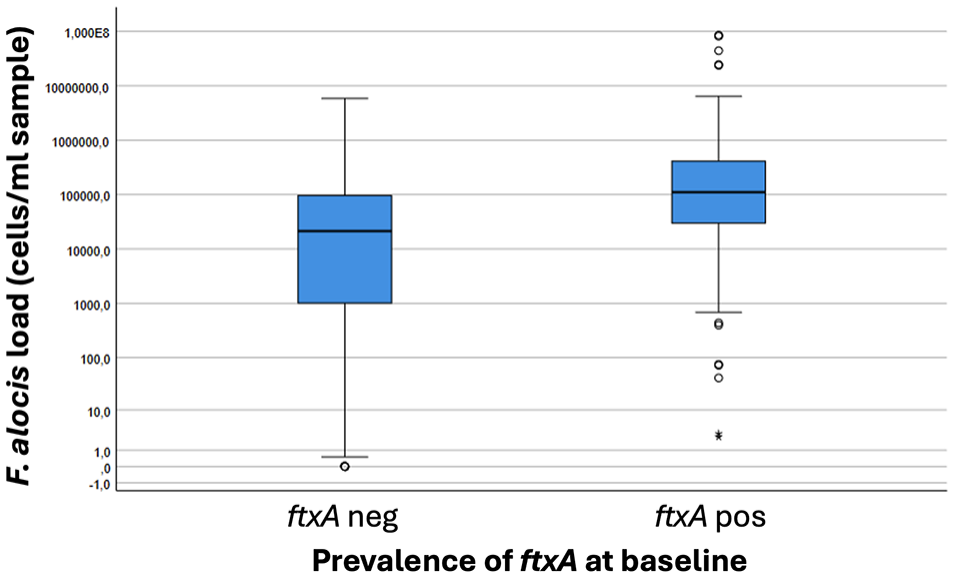
Boxplot illustrating loads of *F*. *alocis* in pooled subgingival plaque samples from carriers of this bacterium (n=457) correlated to presence (positive; pos) or absence (negative; neg) of the *ftxA* gene. *P*<0.001, *ftxA* pos vs *ftxA* neg.

**Figure 4:**
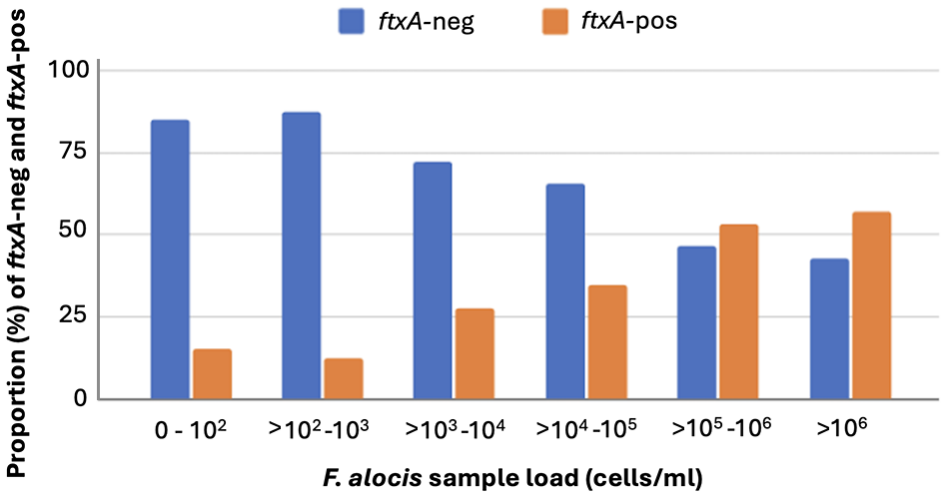
Enhanced proportion of *ftxA*-positive (-pos) compared to *ftxA*-negative (-neg) *F. alocis* upon gradually increasing loads (cells/ml) of this bacterium present in the subgingival plaque samples.

The prevalence of the *ftxA* gene in the analyzed samples at baseline showed a total of 37.2% (*i.e*., of those 486 that were *F. alocis*-positive; ≥10^2^ cells/ml sample) (**Table 1**). Interestingly, the samples analyzed positive for the *ftxA* gene were revealed to have significantly (*p*<0.001) higher loads of *F. alocis* cells/ml in the pooled subgingival plaque samples compared to those that were *F. alocis*-positive but were detected as *ftxA*-negative (**Figure 3**). The proportion of *ftxA*-positive relative to *ftxA*-negative *F. alocis* was also markedly enhanced upon gradually increasing loads of this bacterium present in the samples (**Figure 4**). Taken together this supports the notion that *F. alocis* carrying *ftxA* may exhibit enhanced fitness in the subgingival environment.

### 3.4 Carriers of *ftxA*-positive *F. alocis* exhibits enhanced risk for CAL progression

As summarized in **Table 1** of the *ftxA*-positive samples, 39 out of 170, *i.e.*, 22.9% exhibited CAL. At the follow up, 130 (35.8%) were positive for *ftxA*, and 48.5% of these exhibited CAL. Of the 130 individuals positive for *ftxA*, 38.5% also showed progression of CAL. Moreover, as summarized in **Table 2**, carriers of *ftxA*-positive *F. alocis* showed a significantly increased prevalence of CAL at follow up (OR=1.77; *p*=0.011), but not at baseline (OR=1.38; *p*<0.180). There was also a significant association between progression of CAL and presence of *ftxA* (OR= 1.96; *p*<0.04).

### 3.5 Synergism between *F. alocis* and non-JP2 genotype *A. actinomycetemcomitans* in promoting CAL progression

**Figure 5:**
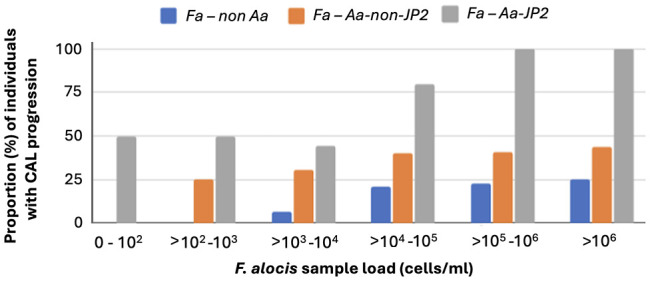
Synergism between *F. alocis* and non-JP2 genotype of *A. actinomycetemcomitans* in promoting CAL progression also at low sample loads. The diagram illustrates the proportion (%) of individuals with CAL progression related to *F. alocis* sample load (cells/ml), and whether the individuals also carried (non-JP2 red bars; JP2 grey bars) or were not carrying (blue bars) *A. actinomycetemcomitans*, respectively.

To assess possible synergism between *F. alocis* (Fa) and *A. actinomycetemcomitans* (*Aa*), we compared the proportions of individuals with CAL progression between carriers of Fa, but not *Aa*, and carriers of Fa and non-JP2 Aa, respectively (**Figure 5**). This revealed that in the absence of *A. actinomycetemcomitans*, *F. alocis* was required to be present in high quantities to promote CAL progression. Interestingly, on the other hand, if *F. alocis*-positive individuals also carried the non-JP2 genotype of *A. actinomycetemcomitans*, there was a synergistic effect enabling the advancement of CAL even at low loads of these bacterial species. Moreover, there was an apparent distinctive influence of the JP2 genotype of *A. actinomycetemcomitans*, consistant with an ability to drive CAL progression regardless of if *F. alocis* was present or not (**Figure 5**).

## 4 Discussion

In the present work, we have used qPCR and PCR, respectively, to characterize the prevalence and carriage loads of the emerging oral pathogen *F. alocis*, and its RTX-toxin encoding *ftxA* gene, in subgingival plaque specimens, sampled at baseline from a longitudinal cohort of Ghanaian adolescents (n=500) (Höglund Åberg et al., 2014; Höglund Åberg et al., 2012). The obtained results were thereafter compared with the recorded CAL and CAL progression data, collected at baseline and at the two-year follow up of the Ghanaian cohort, respectively (Höglund Åberg et al., 2014; Höglund Åberg et al., 2012). The rationale for using this cohort in the present study was that this collection is unique due to being linked to clinical longitudinal data showing attachment loss progression over a two-year time, and it was therefore very suitable for analyzing associations between *F. alocis* carriage, presence of the *ftxA* gene, and periodontal disease progression. Our results revealed that carriers of *ftxA*-positive *F. alocis* typically exhibited higher loads of the bacterium. Moreover, high carriage loads of *F. alocis*, and presence of the *ftxA* gene were two factors both associated with an enhanced prevalence of CAL progression.

The high proportion of individuals with CAL, and CAL progression at the follow up, in the Ghanaian cohort can be partly explained by the presence of *A. actinomyctemcomitans* at baseline, especially the high proportion (8.8%) of individuals carrying the highly leukotoxic JP2 genotype (Höglund Åberg et al., 2014). However, *A. actinomyctemcomitans* cannot itself explain all cases of CAL, and CAL progression in this cohort. One reason for assessing *F. alocis* and its *ftxA* in relation to CAL in the present work was that there appears to be a synergism between *F. alocis* and *A. actinomycetemcomitans* in active periodontal disease, based on observations, such as that *A. actinomycetemcomitans* could stimulate *F. alocis* accumulation, albeit that this effect appeared to be dependent on the strain of *F. alocis* it interacted with (Fine et al., 2013; Wang et al., 2013). Evidently, isolates of *A. actinomycetemcomitans* from the oral cavity are also heterogeneous with respect to their expression levels of LtxA and production of fimbriae (Haubek, 2010; Rylev et al., 2011), and as suggested (Wang et al., 2013), different strains of *A. actinomycetemcomitans* might therefore also exhibit different patterns of interaction with *F. alocis*.

Indeed, the results from our present work would support a scenario where CAL progression is further promoted upon simultaneous presence of *F. alocis* and a non-JP2 genotype of *A. actinomycetemcomitans*. This would be consistent with the notion that there may occur genotype-specific synergy between *F. alocis* and *A. actinomycetemcomitans*. Mechanism(s) for such interbacterial interactions between these two species, and whether it could be a dependence on FtxA, are yet unknown, but could potentially include interplay based on reduction of the oxidative environment, which evidently was conducted via *F. alocis* in synergy between this organism and *Porphyromonas gingivalis* (Mishra et al., 2024). In contrast, CAL progression was not further promoted upon simultaneous presence of *F. alocis* and the highly leukotoxic JP2 genotype of *A. actinomycetemcomitans*, which most likely was a result of an apparent distinctive influence of this genotype to drive CAL progression itself, regardless *F. alocis* was present or not. This would be consistent with the strong leukotoxic activity of the JP2 genotype, as evidenced by the observation that only one bacterial cell was sufficient to lyse the majority of macrophage cells in *in vitro* experiments (Kelk et al., 2008).

Interestingly, *A. actinomycetemcomitans* seems to be outcompeted by *F. alocis* in the deeper periodontal pockets (Dahlén et al., 2014), suggesting that this bacterium can have the capability to adapt and drive the disease process forward in later stages, in the absence of *A. actinomycetemcomitans*. Concomitantly, we recently discovered a possible correlation between deep periodontal pockets at the two-year follow up of the Ghanaian cohort, presence of the *ftxA* gene, and a high quantity of *F. alocis* (Razooqi et al., 2022). As the relatively newly discovered FtxA toxin of *F. alocis* belongs to the same family of RTX proteins as the *A. actinomycetemcomitans* leukotoxin, LtxA (Oscarsson et al., 2020; Linhartova et al., 2010), we hypothesized that high *F. alocis* levels, and carriage of *F. alocis* encoding *ftxA*, might correlate to CAL and to CAL progression in the cohort. Indeed, our present results supported the idea that carriers of *ftxA*-positive *F. alocis* typically exhibited higher loads of the bacterium. Whether this depends on mechanism(s) directly executed by FtxA is not known, but as presence of the *ftxA* gene as well as high carriage loads of *F. alocis* were two factors both associated with an enhanced prevalence of CAL progression, we suggest that FtxA represents a factor promoting the disease progression, at least in younger individuals in these ages.

## 5 Conclusions

Carriers of *ftxA*-positive *F. alocis* typically exhibited higher loads of the bacterium. Moreover, high carriage loads of *F. alocis* and the presence of the *ftxA* gene were two factors that were both associated with an enhanced prevalence of CAL progression. Interestingly, CAL progression appeared to be further promoted upon simultaneous presence of *F. alocis* and non-JP2 genotype of *Aggregatibacter actinomycetemcomitans*. Taken together, our present findings are consistent with the notion that *F. alocis* and its *ftxA* gene promote CAL during periodontal disease. Hence, *ftxA* appears to have the potential to be used as a PCR-based gene marker for the identification of *F. alocis* that are more prone to promote disease progress. This finding might be of importance in the risk assessment in young individuals and is therefore a relevant discovery for development of new diagnostic tools and/or treatment strategies.

## Data availability statement

The raw data supporting the conclusions of this article will be made available by the authors, without undue reservation.

## Ethics statement

The studies involving humans were approved by Medical Research, University of Ghana (IRB 000 1276), and from the local Ethical committee of Umeå University, Sweden (Dnr 2010-188-31M). The studies were conducted in accordance with the local legislation and institutional requirements. The human samples used in this study were acquired from primarily isolated as part of your previous study for which ethical approval was obtained. Written informed consent for participation was not required from the participants or the participants’ legal guardians/next of kin in accordance with the national legislation and institutional requirements.

## Author contributions

ZR: Formal analysis, Investigation, Methodology, Supervision, Validation, Visualization, Writing – original draft, Writing – review & editing. IT: Formal analysis, Investigation, Validation, Writing – original draft, Writing – review & editing. CHÅ: Writing – review & editing, Resources, Supervision. FK: Resources, Writing – review & editing. RC: Conceptualization, Formal analysis, Investigation, Methodology, Resources, Validation, Writing – review & editing. DH: Resources, Writing – review & editing. AJ: Conceptualization, Data curation, Formal analysis, Funding acquisition, Investigation, Methodology, Project administration, Resources, Supervision, Validation, Visualization, Writing – original draft, Writing – review & editing. JO: Conceptualization, Data curation, Formal analysis, Funding acquisition, Investigation, Methodology, Project administration, Resources, Supervision, Validation, Visualization, Writing – original draft, Writing – review & editing.
